# Ovarian Cancer Exosomes Trigger Differential Biophysical Response in Tumor-Derived Fibroblasts

**DOI:** 10.1038/s41598-020-65628-3

**Published:** 2020-05-26

**Authors:** Amy H. Lee, Deepraj Ghosh, Nhat Quach, Devin Schroeder, Michelle R. Dawson

**Affiliations:** 1 Brown University, School of Engineering, Center for Biomedical Engineering, Providence, RI 02912 USA; 2 Brown University, Department of Molecular Pharmacology, Physiology, and Biotechnology, Providence, RI 02912 USA

**Keywords:** Cancer microenvironment, Ovarian cancer, Cell adhesion, Cell migration, Multivesicular bodies

## Abstract

Exosomes are cell-secreted microvesicles that play important roles in epithelial ovarian cancer (EOC) progression, as they are constantly secreted into ascites fluids. While cells spontaneously release exosomes, alterations in intracellular calcium or extracellular pH can release additional exosomes. Yet, little is known about how these exosomes compare to those that are continuously released without stimulation and how they mediate cellular activities important in cancer progression. Here, we demonstrate that chelation of extracellular calcium leads to release of chelation-induced exosomes (CI-exosomes) from OVCAR-3 EOC cells. CI-exosomes display a unique miRNA profile compared to naturally secreted exosomes (SEC-exosomes). Furthermore, treatment with CI- and SEC-exosomes leads to differential biophysical and functional changes including, adhesion and migration in EOC-derived fibroblasts that suggest the development of a malignant tumor microenvironment. This result highlights how tumor environmental factors contribute to heterogeneity in exosome populations and how different exosome populations mediate diversity in stromal cell behavior.

## Introduction

Serous epithelial ovarian cancer (EOC) is the most lethal gynecological malignancy with 5-year relative survival rate of 45%^1^. This poor prognosis is largely attributed to the late diagnosis of high grade EOCs and higher incidence of metastasis. EOC cells metastasizing throughout the peritoneal cavity are often associated with high volumes of ascites fluid^2^. Exosomes are found abundantly in the ascites fluid isolated from ovarian cancer patients^3^; yet, little is known about how these extracellular vesicles alter stromal cells in the peritoneum to promote metastasis.

The progression of cancer is a multistep process that involves many cell types communicating through extracellular signals in the tumor microenvironment (TME) and metastatic niche^4^. Carcinoma associated fibroblasts (CAFs) are a major contributor to these malignant tumor stromas^5^. They mediate hallmark cancer cell behaviors by secreting paracrine factors to alter growth and survival, extracellular matrix (ECM) proteins for matrix remodeling, and pro-inflammatory signals important in tumors and tissues^6^. The number of CAFs is increased in high-grade vs. low-grade EOC tumors, ascites fluids, and solid metastases, and heterotypic CAF and EOC cell spheroids are present in ascites fluids from more aggressive cancers^7^.

Exosomes are extracellular lipid vesicles that traffic critical biomaterials across cell membranes^8^. Their cholesterol-rich membranes protect sensitive proteins and nucleic acids from enzymatic degradation to allow for longer circulation times^9^. Exosome exchange influences cancer-related pathways at all levels, from the initial stages of tumorigenesis to acquisition of drug resistance, and in critical processes that mediate tumor metastasis. These nanovesicles guide critical proteins, transcription factors, and miRNAs through complex extracellular environments to impact distant cell signaling pathways important in cancer dissemination^10^. Cancer cell derived exosomes have been implicated in tumor angiogenesis, immunosuppression, and tissue remodeling to support tumor progression^11^. Exosomes have also been shown to direct organ-specific metastasis by fusing exosome integrins found in their cholesterol-rich membranes with target cells in tissues forming a pre-metastatic niche for cancer cells to follow^12^.

Exosomes are continuously secreted through the endosomal pathway; however, their release can also be triggered by a multitude of stimuli^13,14^. Previous studies demonstrated that culturing cells at low pH to mimic the acidic conditions in the TME significantly increased exosome secretion^13,15^. In addition, increasing the intracellular calcium concentration or the expression of calcium-dependent membrane proteins enhanced exosome secretion^14^. However, little is known about how exosomes secreted spontaneously (SEC-exosomes) compare to exosomes released in response to changes in calcium levels. It is well documented that spikes in intracellular calcium lead to rapid secretion of exosomes^14,16^. This elevated cytosolic calcium stimulation altered the content and increased the amount of secreted exosomes^17,18^. Specifically, calcium stimulation resulted in increased pro-angiogenic factors and plasma membrane lipid raft proteins in secreted exosomes^18,19^. However, the role of extracellular calcium in exosome secretion is not as clearly understood.

We demonstrate that chelation of extracellular calcium using EDTA (Ethylenediaminetetraacetic acid) stimulates the release of a sub-population of exosomes that we refer to as chelation-induced exosomes (CI-exosomes). EDTA is a chelating agent that has been administered intravenously as an ingredient in anti-cancer therapies^20^. Chelation is also used to combat metal ion toxicities^21^. Furthermore, reduced blood serum calcium levels coincide with increased exosome production in pathological conditions such as preeclampsia^22,23^. Thus, it is pivotal to investigate the unique population of exosomes that are released upon extracellular calcium chelation.

Using high-throughput molecular and biophysical assays, we investigated how CI-exosomes functionally differ from SEC-exosomes isolated from the same cancer cells. It is well documented that CAFs and cancer cells use exosome exchange as a cross-talk mechanism^24^. Furthermore, it is established that CAFs play a critical role in reorganizing the ECM of the TME, allowing cancer cells to more easily disseminate from the primary site^25^. We show that CI-exosomes and SEC-exosomes harvested from EOC cells alter the biophysical properties of patient-derived CAFs, inducing more aggressive CAF phenotypes. Both exosome populations altered cytoskeletal fiber and focal adhesion organization to affect the morphology of CAFs. These exosome populations also enhanced random and directional motility of CAFs; however, adhesion strength was only enhanced in CAFs treated with CI-exosomes. Additionally, we observed increased spreading in CAFs treated with CI-exosomes. Furthermore, immunoblot results demonstrated that CI- and SEC-exosomes exhibit different surface marker characteristics. Differences in molecular exosome content were further corroborated through our miRNA microarray analysis, which showed CI- and SEC-exosomes express different levels of miRNAs that have implications on cell adhesion and mechanotransduction. These functional and molecular driven results not only elucidate how exosomes are key regulators in mediating the TME, but also the importance of different exosome populations in mediating these tumor-related responses.

## Results

### Isolation and characterization of exosome populations

**Figure 1:**
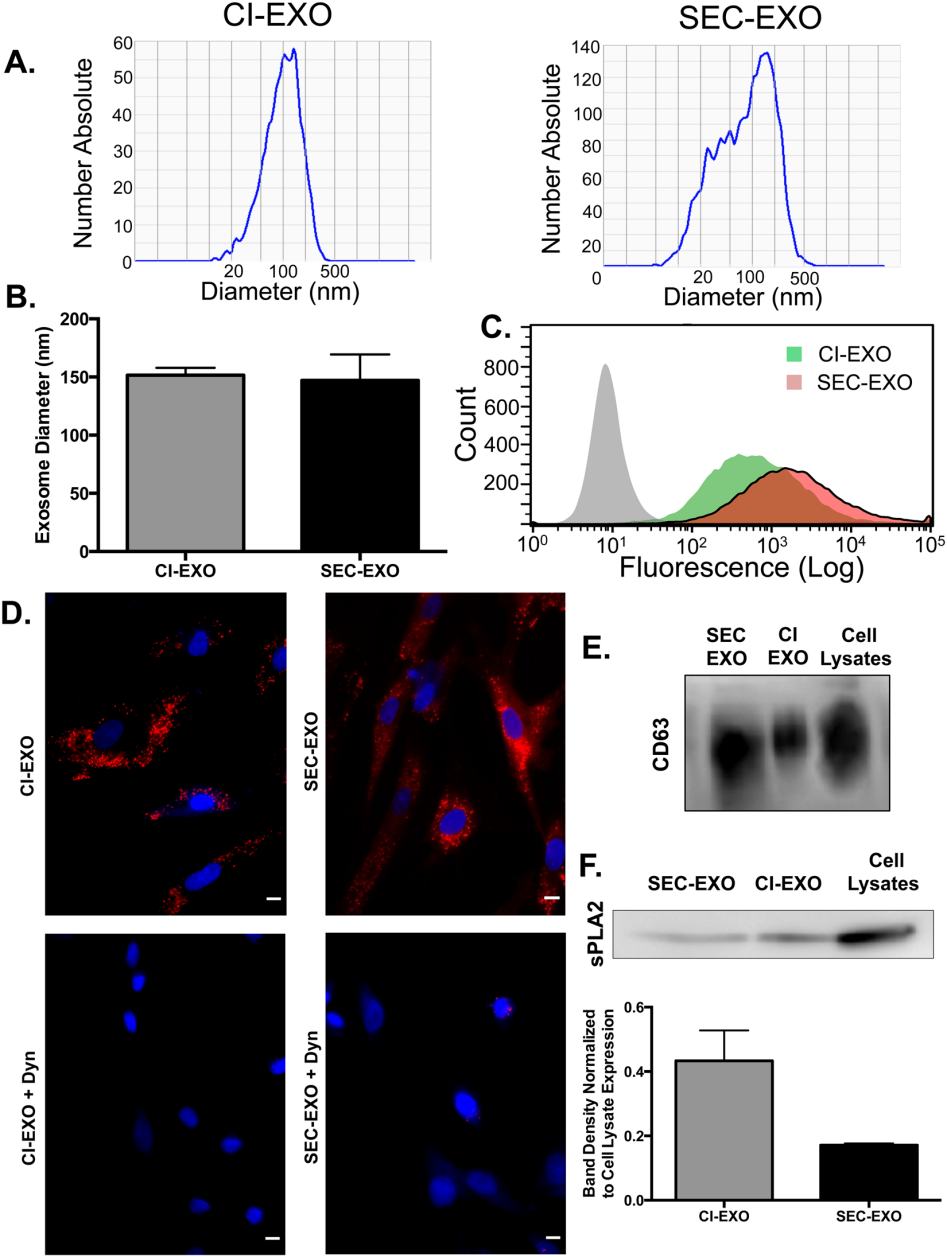
Characterization of OVCAR-3 CI-exosome and SEC-exosome Populations. CI- and SEC-exosomes were collected using standard ultracentrifugation protocols. Quantification of exosome (**A**) diameter and (**B**) size distribution was performed using dynamic light scattering. Although average diameter was similar, the size of SEC-exosomes compared to CI-exosomes was more distributed. (**C**) Flow cytometry histograms of fluorescence intensity for untreated fibroblasts (gray area) and fibroblasts treated with DiI labeled exosomes (green and red areas) for one hour. The rightward histogram shift showed that fibroblasts uptake either population of exosomes. (**D**) Untreated and Dynasore treated CAFs were treated with DiI labeled exosomes for 24 hours at 37 °C. CAFs were then stained with DAPI and merged with 10X magnification. Representative fluorescent images show fibroblast uptake DiI labeled exosomes (top panels) is diminished upon treatment with 10 nM Dynasore (bottom panels). *Scale Bar:* 10 μm. Immunoblots probing for (**E**) CD63 and (**F**) sPLA2 expression in equal number of exosomes. CI-exosomes displayed a higher expression of sPLA2 compared to SEC-exosomes. Expression was normalized to cell lysate expression. Full-length blots are presented in Supplement Fig. 5.

SEC-exosomes were harvested from OVCAR-3 cell conditioned media using ultracentrifugation^26^. OVCAR-3 cells were then washed and treated with EDTA, an extracellular calcium chelator, to release CI-exosomes. The CI-exosomes were also isolated using ultracentrifugation^26^. Exosome diameters were determined using dynamic light scattering (DLS). The average diameters of CI-exosomes and SEC-exosomes were comparable—ranging from 130–150 nm (Fig. 1A); however, variation in exosome diameters was reduced for CI (151.6 ± *6.38 nm*) in comparison to SEC (147.13 ± *22.36 nm*) (Fig. 1A,B).

Next, we confirmed that CAFs rapidly internalize either exosome population using a combinatorial approach of flow cytometry and fluorescence microscopy (Fig. 1C,D). Flow cytometry and fluorescence microscopy highlight exosome uptake at short (1-hour) and long (24-hour) time points, respectively. We also sought to determine if endocytosis was primarily responsible for this internalization. Therefore, we treated fibroblasts with Dynasore, a dynamin inhibitor, to block the endocytic pathway^27^. Fibroblasts treated with 10 nM Dynasore were no longer able to uptake exosomes as effectively, suggesting that endocytosis could be a primary mechanism for internalization (Fig. 1D).

To further characterize CI-exosomes, we investigated differences in surface protein expression between these exosome populations. We ran immunoblots probing for CD63 (Fig. 1E) and secretory phospholipase A2 Group IIA (sPLA2) (Fig. 1F). Phospholipases, including sPLA2, are proteins that are found in the lipid rafts on cholesterol-rich cell and exosome membranes^28,29^. Western blot results showed that both populations of exosomes positively expressed sPLA2 and CD63. Interestingly, CI-exosomes expressed higher levels of sPLA2 compared to SEC-exosomes, suggesting that CI-exosomes may be sequestered in lipid rafts. Together, these data reveal that chelating extracellular calcium elicits the release of a subpopulation of exosomes that exhibit varying physical and molecular characteristics.

### Comprehensive differences in miRNA expression in exosome populations

**Figure 2:**
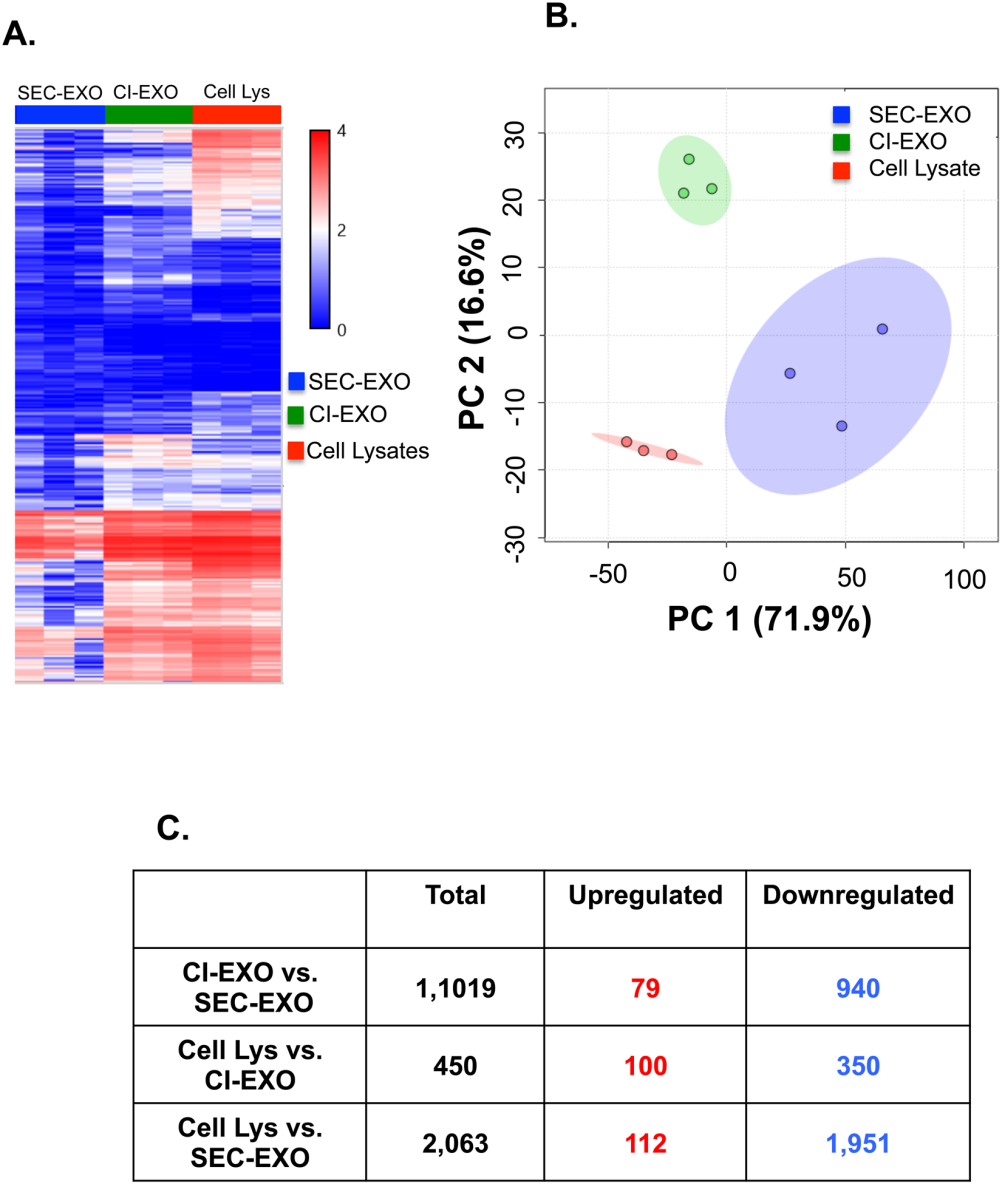
Exosome miRNA Profiling. miRNA from CI- and SEC-exosomes and OVCAR-3 cell lysates (serving as a control) were collected and analyzed. (**A**) Hierarchical clustering analysis and (**B**) PCA mapping were performed for CI-exosome, SEC-exosome, and cell lysate samples. For the PCA plot (cell lysates—red, CI-exosome—green, and SEC-exosome—blue), each point represents a biological sample, the *x*-axis represents first principal component, and the *y*-axis represents second principal component. In our miRNA data, the first two principal components account for 71.9% and 16.6% of the total variability in miRNA expression, respectively. The first two principal components explain 88.5% of the variance. The observed principal components consequently led to separate groups between exosomes and cell lysates. (**C**) Table representing the number of miRNAs that are differentially upregulated and downregulated between population of exosomes and cell lysates.

We next sought to determine if either population of exosomes presented unique miRNA profiles. This is important because exosome-secreted miRNAs play crucial roles in regulating post-transcriptional gene expression important in cancer progression^30,31^. Therefore, we performed microarray analysis to determine the miRNA content in CI- and SEC-exosome populations, along with OVCAR-3 cell lysates that served as a miRNA control. From the total 2,578 human miRNA probes the genechip miRNA 4.0 array identified, we limited our screening to miRNAs with log2 fold differences in expression levels and p-values <0.05 between CI- and SEC- exosomes. Hierarchical clustering and two-dimensional principal component analysis (PCA) of CI- and SEC- exosomes (Fig. 2A,B) suggest specific differences in miRNA content between exosome populations and cell lysate. PCA also showed decreased heterogeneity in CI-exosome compared to SEC-exosome miRNA content. We then examined the number of miRNAs that were differentially expressed between each group (Fig. 2C). This analysis showed the greatest overlap in miRNA content between CI-exosomes and cell lysate (with only 450 differentially expressed miRNAs). This was in contrast to SEC-exosomes and cell lysate, which had the largest variation in miRNA content with 2,063 differentially expressed miRNAs. We also identified 1,019 miRNAs were differentially expressed between CI- and SEC- exosomes; this included 79 upregulated and 940 downregulated miRNAs.

### miRNA regulation of mechanosensitive pathways important in cancer progression

**Figure 3:**
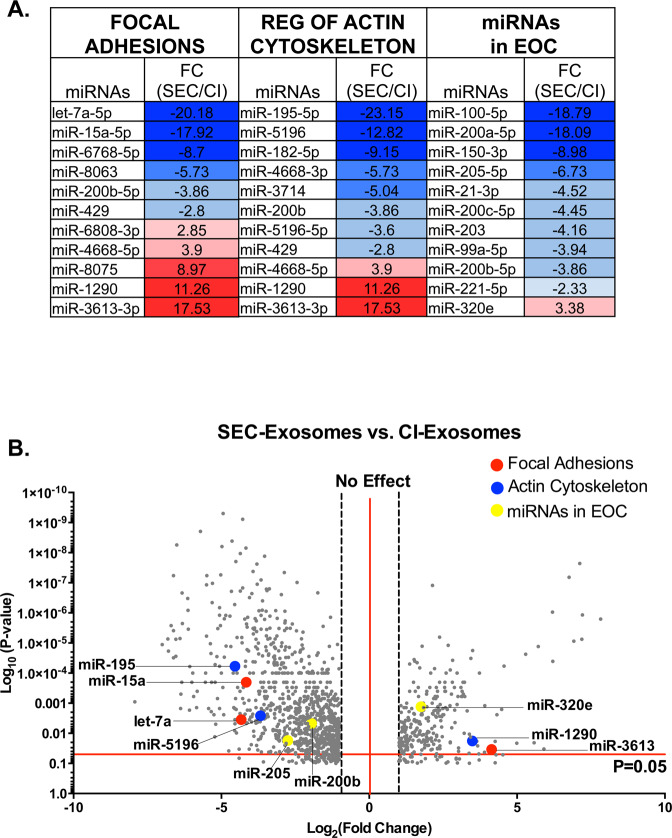
Exosome miRNA Associated Cancer and Mechanosensitive Pathways. Comprehensive miRNA microarray analysis between SEC-exosome vs. CI-exosome showed that various miRNAs were differentially up- and downregulated between exosome populations. (**A**) Analysis was used to determine Log2 fold changes after FDR correction of representative, specific miRNAs that are associated to focal adhesions, actin cytoskeleton, and epithelial ovarian cancer. The compiled list of associated miRNAs was analyzed using DIANA TOOLS. Several miRNAs that were highly regulated overlapped in presented pathways. (**B**) A representative list of differentially regulated miRNAs between exosome populations were plotted in the volcano plot (gray) and examples of up- or downregulated miRNAs are highlighted for specific pathways, including focal adhesions (red), actin cytoskeleton (blue), and miRNAs in EOC (yellow).

Functional analysis of miRNAs was performed using DIANA miRPath v3.0 and KEGG database to identify mRNA targets and gene regulatory networks affected by differentially expressed miRNAs (Fig. 3). These short non-coding miRNAs target multiple mRNAs to regulate gene expression through cleavage and/or repression of translation^32^; thus, pathway analysis is important in understanding the complex interactions between coding and non-coding RNAs in regulating gene networks. Our pathway analysis revealed increased expression of miRNAs associated with mechanosensitive cellular responses, such as focal adhesions dynamics, actin cytoskeleton, pathways in cancer, and EOC progression (Fig. 3, Sup. Table 1). We examined miRNA regulation between each pair [CI-exosomes vs. cell lysates (Sup. Figure 2A), SEC-exosomes vs. cell lysates (Sup. Figure 2B), and SEC-exosomes vs. CI-exosomes (Fig. 3)]. Both exosome populations exhibited different miRNA expression compared to cell lysates with the largest variation in SEC-exosomes (Sup. Figure 2B). SEC-exosomes also displayed a greater number of downregulated miRNAs with larger fold differences in miRNA expression compared to cell lysates.

Next, we performed more in-depth analysis of select miRNAs involved in mechanosensitive cellular responses in SEC- vs. CI- exosomes. We report expression fold changes in key miRNAs in these pathways (Fig. 3A,B). We found several overlapping miRNAs involved in these mechanosensitive pathways, including miR-429, miR-200a-c, and miR-1290. SEC-exosomes had a reduced expression of miR-429 compared to CI-exosomes. This is significant because miR-429 plays a critical role in EOC progression^33^; downregulation of miR-429 induces tumorigenesis and patients with lower levels of miR-429 exhibit reduced overall survival^34^. We further compiled a list of miRNAs that serve as biomarkers for poor prognosis in ovarian cancer (Fig. 3A). In particular, the expression of the miR-200 family has been associated to many ovarian cancers and has been reported to serve as a biomarker of poor survival and early relapse in stage I ovarian cancers^35–37^. We observed that CI-exosomes have an elevated expression of miR-200a-c compared to SEC-exosomes. Taken together, our miRNA array reveals that CI-exosomes and SEC-exosomes exhibit unique miRNA expression profiles differentially regulating key cytoskeletal and adhesion pathways implicated in cancer progression.

### Exosome treatments induce actin cytoskeletal remodeling

**Figure 4:**
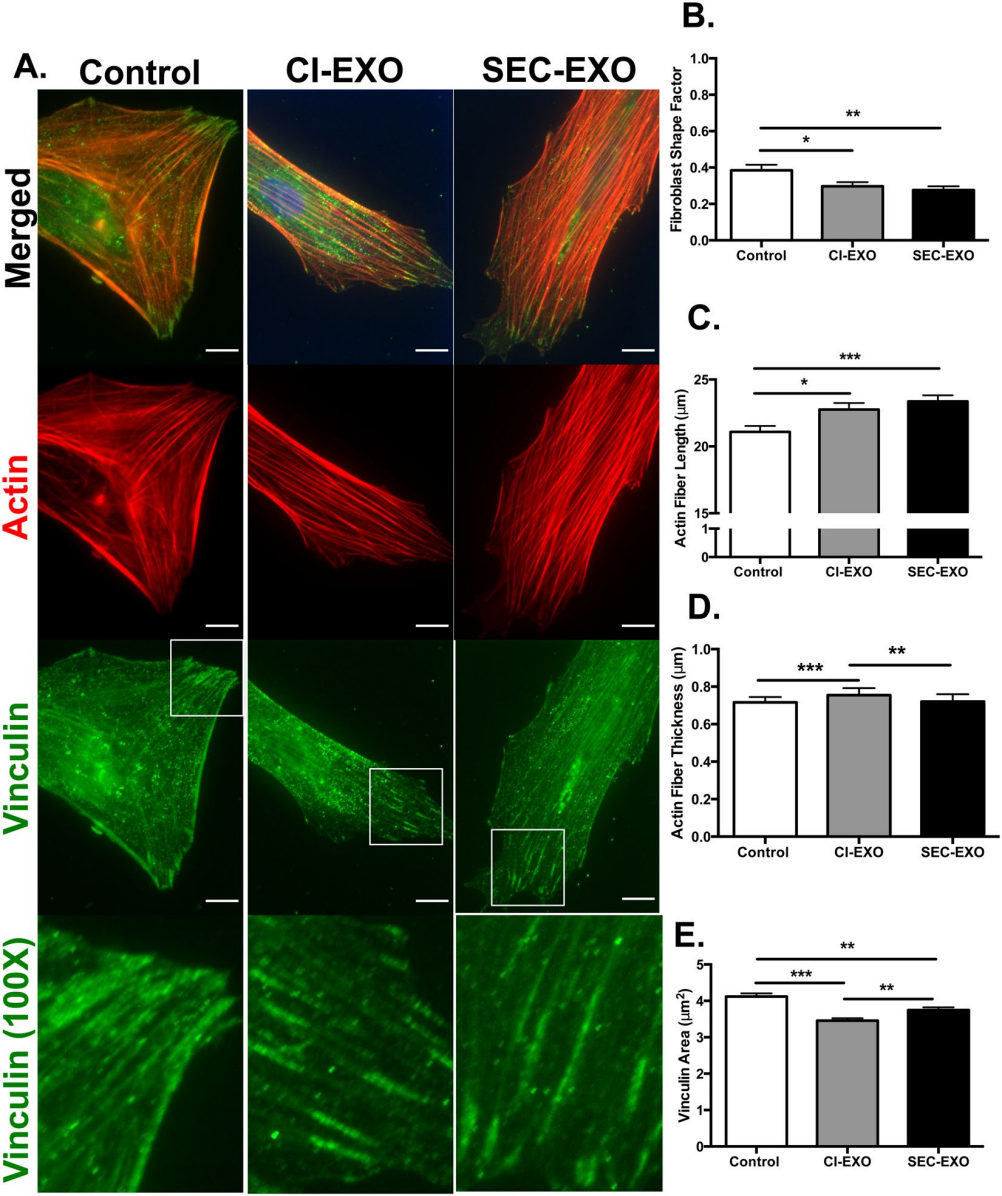
Immunocytochemistry and Quantification of Actin/Vinculin. (**A**) 40X images and 100X zoomed-in images of boxed white regions of CAFs (Control, treated with CI-exosome, and SEC-exosome for 24 hours) were stained for actin (red) and vinculin (green) to determine actin and focal adhesion protein organization. *Scale Bar:* 10 μm. (**B**) CAF shape factor, (**C**) actin fiber length, (**D**) actin fiber width, and (**E**) vinculin area were analyzed for each exosome condition (N = 3). CAF morphology was measured using ImageJ, actin fiber lengths and widths were measured using CT-Fire, and vinculin area was measured using Cell Profiler. Exosome treated CAFs revealed more elongated morphology. Quantification of actin fiber parameters showed that both exosome populations induced longer actin fibers, whereas CI-exosomes induced the development of thicker actin fibers. Vinculin area decreased upon exosome treatment and dispersed at CAF edges. Statistics calculated using Student’s t-test and reported as values +/− SEM. *p < 0.05 **p < 0.005, ***p < 0.001.

Since tumor-derived exosomes have been implicated in cancer progression by altering stromal cell behavior in tissue and tumor microenvironments, we next sought to determine how CI- and SEC- exosomes affect the biophysical properties of resident fibroblasts^38^. Fibroblast differentiation into CAFs has been associated with morphological elongation and actin stress fiber formation so we examined these biophysical properties^39,40^. Our patient-derived CAFs naturally exhibited a spindle-like morphology consistent with other literature;^5^ however, after 24-hour exosome treatment, many of these CAFs became even more elongated (Fig. 4A). The cell shape factor was quantified in ImageJ, where cell shape factors of 0 and 1 represent a line and circle, respectively. Both CI- (p = 0.0254) and SEC- (p = 0.0047) exosome treatments resulted in morphological elongation compared to control as indicated by significant reductions in their CAF shape factors (Fig. 4B). There was no significant difference in CSFs between exosome treatment groups (p = 0.443).

Because we quantified morphological changes in CAFs treated with CI- or SEC-exosomes, we projected that cytoskeletal fibers and focal adhesion proteins would reorganize to mechanically support changes in cell shape^41^. CAFs were stained for filamentous actin using Phalloidin and focal adhesions using vinculin antibody (Fig. 4A). Treatment with either exosome population resulted in increased alignment of actin filaments and the formation of long actin stress fibers (CI-exosomes, p = 0.0125; SEC-exosomes, p = 0.0005; CI-exosomes vs. SEC-exosomes, p = 0.3583). The longest actin stress fibers were seen in CAFs treated with SEC-exosomes (Fig. 4C). This result was consistent with their lower fibroblast shape factor, and likely indicates more polarized actin cytoskeleton after exosome treatment. CAFs treated with CI-exosomes also developed longer actin stress fibers; however, these actin stress fibers were also thicker (CI-exosomes vs. Control, p = 0.0007; CI-exosomes vs. SEC-exosomes, p = 0.0082) (Fig. 4D), suggesting that CI-exosome treatment induces actin filament bundling. These results reveal that CAF treatment with either exosome population induces more polarized actin cytoskeletal structures with differences in cytoskeletal organization.

Focal adhesions are dynamic integrin-based adhesion complexes that anchor the actin cytoskeleton to the ECM; they play a critical role in transferring environmental stimuli to the cell to alter cell shape, adhesion, and motility^42^. Based on the observed changes in cytoskeletal actin, we hypothesized that exosome treatment would alter the distribution of focal adhesions complexes. In CAFs treated with either exosome population we observed focal adhesions that more clearly aligned with actin stress fibers. These focal adhesions were often localized to the ends of actin stress fibers after exosome treatment (Fig. 4A). Using Cell Profiler, we quantified the size of focal adhesions based on the area of vinculin punctae. We observed smaller focal adhesions in fibroblasts treated with either exosome population (CI-exosomes vs. Control, p = 0.0001; SEC-exosomes vs. Control, p = 0.0011; CI-exosomes vs. SEC-exosomes, p = 0.0046) (Fig. 4E). This may indicate that untreated CAFs have more stable actin cytoskeleton with larger and more mature focal adhesions; whereas, exosome treatment leads to more dynamic actin cytoskeleton with smaller and more nascent focal adhesions.

### Exosome treatments enhance random and directional migration

**Figure 5:**
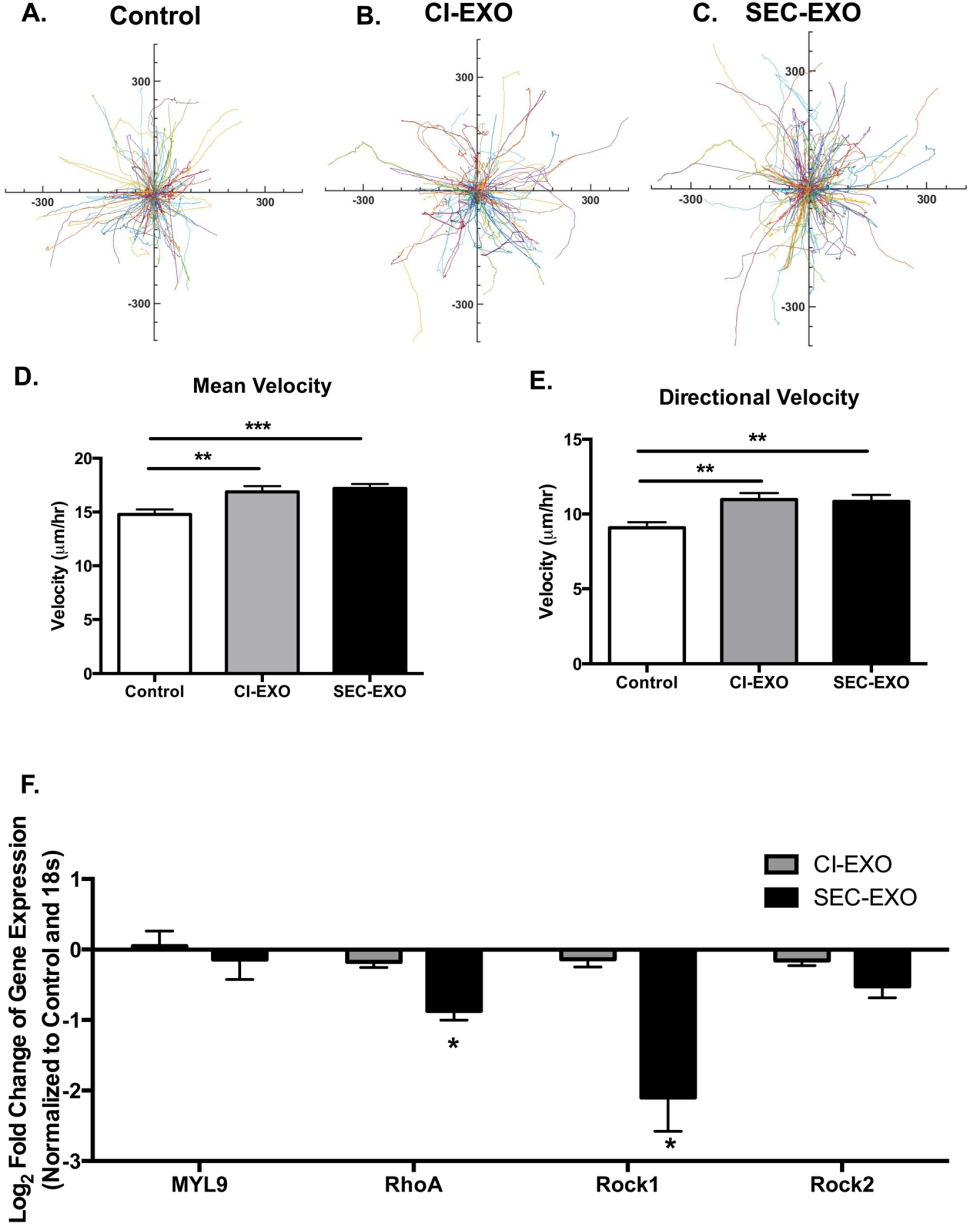
Exosome Treatment Alters CAF Motility Profiles. (**A–C**) Representative random traces of control and exosome treated CAF migration (length scale in nm). Fibroblasts were pre-treated with exosomes for 24 hours in serum free media before tracking their migration for 12 hours at 10-minute intervals on FN-coated surfaces. Mean and directional velocities were measured by tracking Hoechst-labeled nuclei. (**D**) Random and (**E**) directional velocities were measured to determine how exosome treatment varies motility parameters relative to control groups. For motility studies N > 400 cells for all conditions. Statistics calculated using student’s t-test and were reported as mean +/− SEM. Both exosome populations significantly increased mean and directional velocities. (**F**) Rho-Rock pathway gene expression analysis of CAFs treated with either population of exosomes was examined using qRT-PCR. SEC-exosomes significantly decreased the expression of *RhoA* and *ROCK1*. Gene expressions were normalized to untreated fibroblast (control) and 18S expression. Statistics calculated using Student’s t-test. *p < 0.05 **p < 0.005, ***p < 0.001.

Previous studies demonstrated increased migration of exosome treated cells^43–45^. Luga *et al*. showed that exosomes activated Wnt-signaling pathways that led to cells developing more protrusions and cells migrating faster^44^. Additionally, McAtee *et al*. demonstrated cancer-cell derived exosomes enhanced the migration of stromal cells through Boyden chamber wells coated with collagen^46^. Thus, we next investigated how our exosome treatments would affect CAF motility. Fibroblasts grown on fibronectin (FN)-coated surfaces were pre-treated for 24 hours with either exosome population; migration profiles were then measured over a 12-hour period. Starburst plots display representative traces of fibroblast migration for control and exosome treated conditions (Fig. 5A–C). The mean (CI-exosome vs. Control, p = 0.0029; SEC-exosomes vs. Control, p = 0.0001; CI-exosomes vs. SEC-exosomes, p = 0.652) and directional (CI-exosomes vs. Control, p = 0.0012; SEC-exosomes vs. Control, p = 0.0022; CI-exosomes vs. SEC-exosomes, p = 0.741) velocities were determined from cell tracking data (Fig. 5D,E). This data shows that exosome treated cells move faster and more directionally than untreated cells (Fig. 5A–E). Increased CAF migration is important in matrix remodeling to enhance tumor growth and spreading of cancer cells.

Actin myosin (actomyosin) contractility plays a critical role in actin cytoskeletal reorganization and cell motility^47^. Thus, we performed gene expression analysis of key molecules involved in this pathway^48,49^. This included myosin regulating protein *MYL9*, Rho family small GTPase *RhoA*, and Rho-associated kinases *ROCK1* and *ROCK2*. *RhoA* and *ROCK* have both been associated with matrix-stiffness induced malignancy ^50,51^ and play key roles in mechanotransduction pathways^52^. We observed similarities in gene expression for CI-exosome treated CAFs and control (Fig. 5F), suggesting that these genes are not responsible for observed biophysical differences in CAFs treated with CI-exosomes. The expression of *RhoA* (p = 0.0460) and *ROCK1* (p = 0.0304) were both significantly reduced in CAFs treated with SEC-exosomes compared to control, suggesting these genes are more important in regulating cytoskeletal alterations in this population. Previous studies demonstrated that CAFs treated with ROCK inhibitor have smaller focal adhesions^53^ and less vinculin expression;^54^ these focal adhesions were less stable resulting in faster migration^54^. Therefore, the reduced *ROCK1* and *ROCK2* expression may contribute to the smaller vinculin punctae and increased migration observed in CAFs treated with SEC-exosomes.

### Exosome treatments result in differences in CAF adhesion strength and spreading

**Figure 6:**
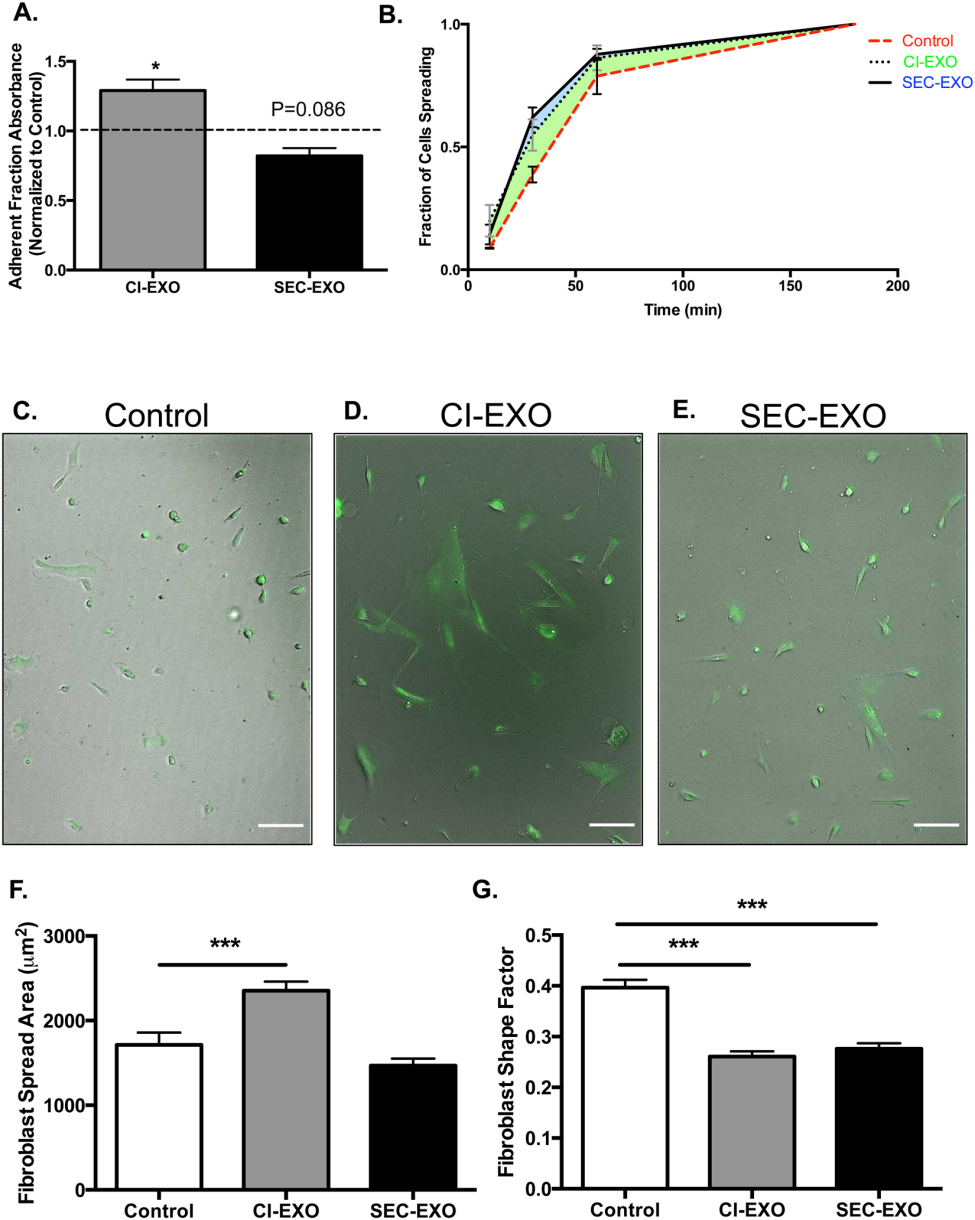
Exosome Treatment Regulates CAF Detachment and Spreading Profiles. CAFs were seeded overnight on FN-coated surfaces. Cells were treated with or without exosomes for 24 hours and detached using a centrifugal force-based assay. (**A**) Adherent fractions of fibroblasts treated with either population of exosomes were normalized to control groups. The dashed line indicates control group. Statistics calculated using Kruskal-Wallis test and were reported as mean +/− SEM. (**B**) To determine the fraction of cells spreading, CAFs were treated with exosomes for 24 hours, detached, and adhered on FN coated surfaces. The number of cells spreading over total number of cells at each time point was reported. (**C–E**) 10X images of calcein-labeled fibroblasts for each condition were taken to analyze cell spread profiles after 12 hours. *Scale bar:* 100 μm. (**F**) Fibroblast spread area and (**G**) shape factor were measured at the 12-hour time point (N = 3). Statistics calculated using Student’s t-test and reported as values +/− SEM. *p < 0.05, ***p < 0.001.

The rapid assembly and turnover of focal adhesions are critical in cancer progression as cells migrate, adhere, and spread on multiple ECM-coated surfaces^42,55^. Focal adhesion complexes also cluster integrins to the cell surface to modulate adhesion strength to promote cell attachment and spreading on ECM-coated surfaces^56^. However, too much adhesion strength from large focal adhesions may limit the rate of focal adhesion turnover and cell migration. Consistent with this idea, less migratory control cells had larger focal adhesions and migrated more slowly than exosome treated cells, which displayed smaller but more diffuse patterns of focal adhesions (Figs. 4A,D; 5A–E). We used a centrifuge-based assay to probe for differences in adhesion strength for control and exosome treated CAFs on FN-coated surfaces. Treatment with CI-exosomes resulted in increased adhesion strength (p = 0.0350); this was surprising since the focal adhesion area per cell was actually diminished (Fig. 6A). However, CAFs treated with CI-exosomes formed thick actin stress fibers with small focal adhesions that were distributed throughout the cells; this pattern of focal adhesions may support Velcro-like interactions between actin stress fibers and the FN-coated surfaces. CAFs treated with SEC-exosomes displayed the lowest adhesion strength (Fig. 6A) (p = 0.086); this was not surprising since these cells exhibited smaller focal adhesions that were often localized to the ends of long actin stress fibers, providing fewer points of attachment with the FN-coated surfaces.

To further investigate this dynamic adhesion process, we used time-lapsed imaging to monitor cell attachment and spreading on FN-coated surfaces. CAFs were treated with exosome populations for 24 hours, then detached, and re-seeded on ECM-coated surfaces; we report the initial adhesion rates and spreading profiles measured at multiple time points over a 12-hour period. Exosome treatment resulted in faster adhesion to FN-coated surfaces; this is based on increased fraction of spread cells at the initial time point (Fig. 6B). The fraction of spread cells was increased in exosome treatment groups until the 12-hour time point when the majority of cells in all groups had adhered (Fig. 6B); this data demonstrates that exosome pre-treatment enhances CAF adhesion rates. Next we probed for morphological differences in CAFs at the 12-hour time point. Interestingly, CAFs treated with CI-exosomes exhibited markedly greater spread areas (p = 0.0004), while SEC-exosome treated CAFs displayed similar spread areas as the untreated CAFs (Fig. 6C–F). This increase in spread area for CI-exosome treated cells could correlate to the increase in fibroblast adhesion strength, suggesting that the focal adhesions in this CAF populations are more stable and can withstand greater detachment forces. Although exosome treatments led to differences in spread area, CAFs treated with either exosome population displayed more elongated phenotypes (Fig. 6G), consistent with more pronounced CAF phenotype.

### Exosomes alter crosstalk between CAFs and Ovarian Cancer Cells

**Figure 7:**
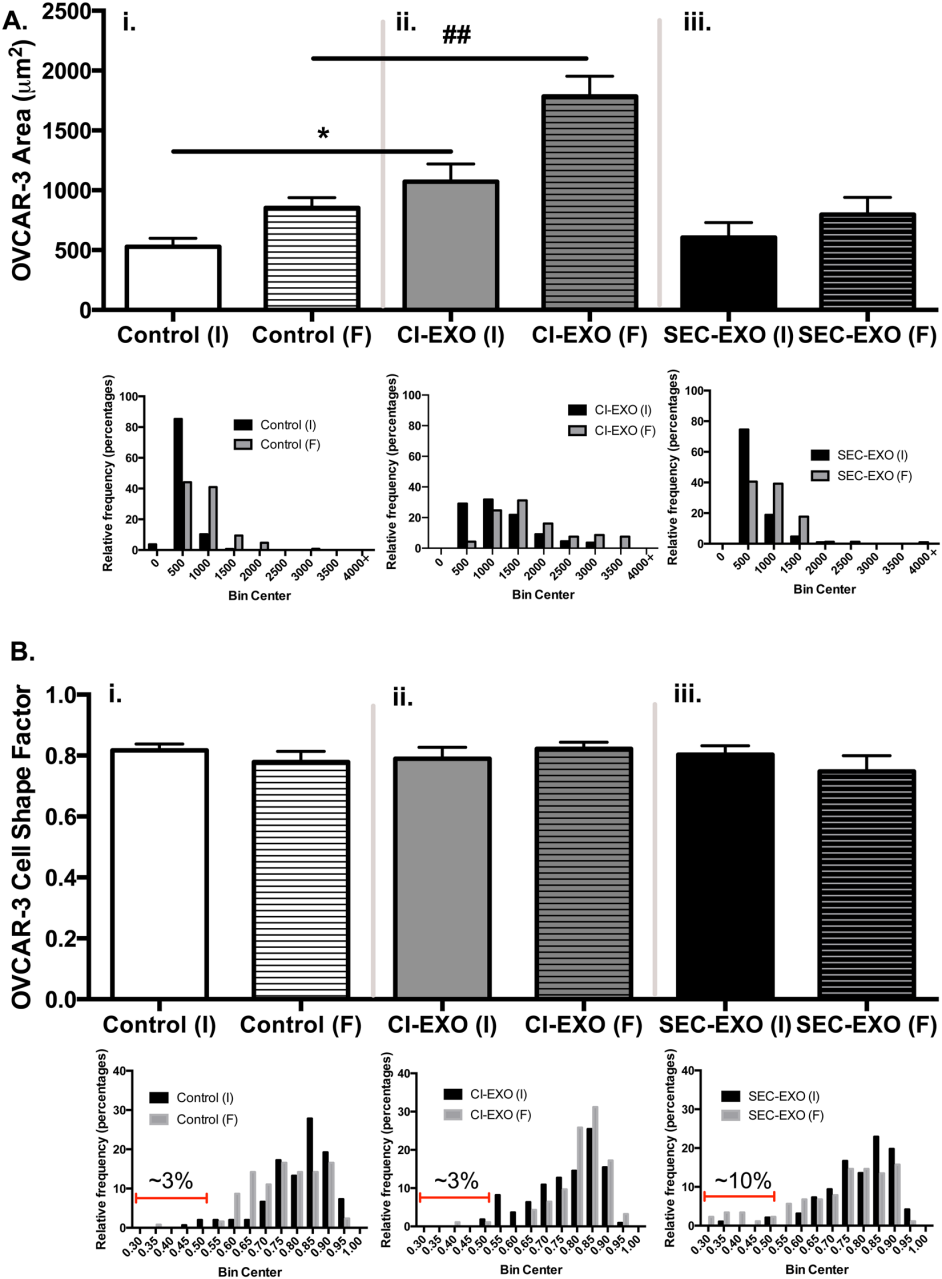
Exosome Treatment in a Coculture System Leads to Differences in Cancer Cell Morphology and Area. Equal number of CAFs and OVCAR-3 were seeded on surfaces and were treated with or without exosomes. Images of coculture model were taken every 30 minutes over a 16-hour period. (**A**) OVCAR-3 area average and distribution were measured at initial (I) and final (F) time points using ImageJ (N = 3). (ii) CI-exosome treatment significantly increased OVCAR-3 spread area compared to (i) untreated spread areas at respective I and F time points. (iii) SEC-exosome treatment did not show any statistically significant change in spread area. (**B**) Average shape factor results show no statistical difference between (ii-iii) exosome treatment and (i) untreated groups at either initial (I) or final (F) time points. Interestingly, histogram distributions showed that (iii) SEC-exosomes led to a small percentage of elongated outlier OVCAR-3 cells at final time points (black) compared to initial points (gray). The highlighted red-gated region in (i-iii) represents OVCAR-3 that had shape factors less than 0.5. Specifically, we quantified that SEC-exosomes induced elongation in approximately 10% of OVCAR-3. Untreated and CI-exosomes induced elongation in approximately 3% of OVCAR-3. Statistics calculated using Student’s t-test and reported as values +/− SEM. *p < 0.05, ^##^p < 0.005.

We previously showed that crosstalk with stromal cells mediates the adhesion and spreading of cancer cells and induces a more elongated mesenchymal phenotype in epithelial cancer cells^57,58^. In co-culture studies with equal numbers of OVCAR-3 cells and CAFs, we further examined how crosstalk between these two cell types is modified by exosome treatment. Time-lapsed microscopy was used to monitor how exosome treatments affect the attachment and spreading of OVCAR-3 cells over a 16-hour period in the presence of CAFs (Sup. Figure 3A–C). The spread area of OVCAR-3 cells increased with time from the initial (I) to final (F) time point for all conditions (Fig. 7A). However, OVCAR-3 cells attached more rapidly and spread more completely in co-cultures treated with CI-exosomes compared to control; this was demonstrated by significant increases in the initial (p = 0.012) and final (p = 0.0036) OVCAR-3 cell spread areas (Fig. 7Ai,ii), respectively. In contrast, treatment with SEC-exosomes did not significantly alter the initial and final spread areas of OVCAR-3 cells relative to control (Fig. 7Ai,iii). We next looked for changes in cancer cell morphology (Fig. 7B). The average cancer cell shape factor was similar for all groups (CSF ~0.8), indicating the majority of OVCAR-3 cells remain round regardless of time or exosome treatment. In part to the heterogeneity in epithelial cancer cells, we used heterogeneity analysis to examine the distribution in cancer cell shape factors for all conditions. In control cells, the distribution in cancer cell shape factors increased with time ((Coefficient of Variation) CV, I = 0.126; CV, F = 0.144), demonstrating the natural heterogeneity in cancer cell morphology after spreading (Fig. 7Bi, Sup. Table 2). The increased variation in cancer cell shape factor after spreading was also seen in co-cultures treated with SEC-exosomes (CV, I = 0.132; CV, F = 0.238) (Fig. 7Biii, Sup. Table 2). In CI-exosome treated cells, cancer cell shape factors were more distributed initially compared to the final time point. At the initial time point, the cancer cells were also much larger than in the other conditions, likely indicating that CI-exosomes prime cancer cells for more rapid adhesion (Fig. 7Aii). Immediately after CI-exosome treatment, we also observe an increase in the percentage of cells with an intermediate morphology (0.55 < CSF < 0.75); however, as these cells continue to spread, their morphology begins to become more round (CV is reduced and CSF increased). Although the majority of OVCAR-3 cells remain round for all conditions, a small number of cells develop a much more elongated phenotype after spreading (CSF < 0.5). The % of more elongated “outlier” cells was increased from ~3% in untreated controls to ~10% in co-cultures treated with SEC-exosomes (Fig. 7Biii). Taken together, our co-culture studies indicate that CI-exosomes more widely promote adhesion and spreading of cancer cells, whereas SEC-exosomes increase the percentage of highly elongated “outlier” cells.

## Discussion

Paracrine interactions between EOC cells and the surrounding tissue microenvironment, with its diversity in cell types and matrix mechanics, play a critical role in directing EOC metastasis^59^. Tumor derived exosomes transfer nucleic acids and proteins between various cell types to mediate cell-cell communication and signaling pathways important in cancer progression. EOC cells are shed from the primary tumor into the peritoneum as individual cells or multi-cellular spheroids and often adhere and metastasize to the omentum, a common site for EOC metastasis^2^. Exosomes shed from the primary tumor into the ascites fluid may play an important role in preparing this niche for EOC metastasis^60^. Here, we demonstrate that an extracellular calcium perturbation, using EDTA, led to the release of a unique population of exosomes, referred as CI-exosomes, which possessed unique miRNA signatures compared to the traditionally studied secreted exosomes, referred as SEC-exosomes. Furthermore, these populations of EOC-derived exosomes elicited more pronounced CAF phenotypes characterized by biophysical and molecular alterations. Importantly, treatment with CI-exosomes resulted in critical differences in adhesion strength, important in mediating critical steps in EOC metastasis.

CAFs are myofibroblast-like cells found abundantly in tumor tissues. They play a central role in tumor growth and matrix remodeling^61^ by increasing matrix deposition, cross-linking, and bundling for increased tissue stiffness^62,63^, activating mechanosensitive signaling pathways important in cancer^64^, and generating force and protease-mediated tracks that cancer cells follow during metastasis^65^. They arise from normal fibroblasts that have been activated by tumor-secreted factors to form CAFs^66^. A CAF-like phenotype is characterized by changes in cytoskeletal architecture, motility, and adhesion patterns, along with increased expression of a-smooth muscle actin (a-SMA), platelet derived growth factor receptor beta (PDGFR-B), and fibroblast activated protein (FAP)^67^. CAFs treated with either population of exosomes increased in FAP and PDGFR-B expression (data not shown). The patient-derived CAFs naturally expressed high levels of a-SMA; therefore, noticeable increases in a-SMA expression were difficult to observe after exosome treatment. However, our data demonstrated undifferentiated lung fibroblasts that had a low basal level of a-SMA showed upregulated expression upon exosome treatment (Sup. Figure 4). In our study we demonstrate that CAFs treated with exosomes isolated from EOC cells, including both CI- and SEC- exosome populations, develop more striking CAF phenotypes. This was demonstrated through changes in the actin cytoskeleton, including increased actin stress fiber formation and alterations in the patterns of focal adhesion proteins (Fig. 6). Increased actin fiber length and bundling are hallmarks of a more activated CAF-phenotype; these structural changes in tumor stromal cells are important in generating aligned tracks in the ECM that allow cancer cells to invade during metastasis^68^.

Dynamic cytoskeletal proteins, such as actin, can also directly affect the turnover of focal adhesions that anchor actin filaments to the ECM^69^. Vinculin orientation has been shown to directly regulate cell polarization, which then dictates the direction of cell migration^70,71^. Consistent with studies that link vinculin orientation and migration, SEC-exosome treatment induced more brush-like pattern of focal adhesions that decorated long actin filaments that were polarized for enhanced cell migration (Fig. 4A, Fig. 5C). Fibroblast treatment with SEC-exosomes also affected the molecular expression of actin myosin contractility genes (including *RhoA* and *ROCK1*) important in cell migration (Fig. 5F); these changes were consistent with previous ROCK inhibitor studies resulting in increased stromal cell migration and wound closure^53,54^.

Fibroblast and cancer cell interactions are particularly important in EOC metastasis since ovarian cancer cells often metastasize through ascites as spheroid aggregates containing stromal cells^7,72^. Fibroblasts in these spheroids have been shown to enhance the survival of cancer cells and guide peritoneal invasion during metastasis^7^. Dynamic adhesion processes regulate the shedding of cancer cells at the primary site, as well as their attachment at the secondary site^73^. We hypothesized that exosome crosstalk between cancer cells and CAFs would also be critical in mediating these dynamic adhesion processes. We show that CAFs treated with SEC-exosomes detach faster and adhere more rapidly to FN-coated surfaces (Fig. 6A,B). The decrease in adhesion strength with the treatment of SEC-exosomes could assist CAFs to readily detach from the primary site while the more rapid adhesion turnover may promote reattachment at the secondary site. CI-exosome treatment strengthened fibroblast adhesion and promoted greater cell spreading to FN-coated surfaces (Fig. 6F), suggesting this exosome population may be important in allowing fibroblasts to firmly attach at the metastatic site. Our data suggests that tumor-derived exosomes would elicit critical biophysical changes in CAFs to guide their tissue interactions through the metastatic process.

Previous studies have illustrated how exosome miRNAs directly affect cell functional properties^74–76^. For instance, the increased expression of miRNA-1290 has previously been associated with enhanced cell motility and invasion^77,78^. Similar to this result, our microarray data show miRNA-1290 is highly expressed in SEC-exosomes. CAFs treated with SEC-exosomes also showed elevated motility (Fig. 3A). Conversely, our microarray results show that SEC-exosomes had lower expression of miRNA-200a-c in comparison to CI-exosomes (Fig. 3A). The miRNA-200 family is used as a diagnostic marker for EOC^36,79^. High grade EOCs often displays elevated levels of miRNA-200, which is associated with increased expression of E-cadherin^80^. Increases in E-cadherin expression in epithelial surface invaginations of the ovary have been shown to induce metaplastic changes and tumor cell proliferation^80^. A previous study has reported that the knockdown of the miRNA-200 family resulted in reduced adhesion with increased motility^81^. Based on the miRNA 200 family expression, our data similarly suggests that SEC-exosomes may be more likely to induce more migratory phenotypes in stromal cells and an epithelial-mesenchymal transition (EMT) response in EOC cells; whereas, the CI-exosomes would be more likely to mediate strong cell adhesions and mesenchymal-epithelial transition (MET) in cancer cells. MET is thought to be critically important in EOC; however, this process is not well understood and remains highly understudied.

To examine the effects of exosome-mediated crosstalk, we investigated the biophysical effects of cells that largely constitute the primary TME: ovarian cancer cells and CAFs (Fig. 7). Our co-culture model showed that the addition of either population of exosomes altered the biophysical phenotype of OVCAR-3 cells (Sup. Figure 3). Specifically, CI-exosomes promoted rapid attachment and spreading of cancer cells; whereas, SEC-exosomes increased the percentage of highly elongated “outlier” cancer cells. Studies have shown cancer cells elongating as a hallmark of EMT. Our co-culture model further suggests that SEC-exosomes could induce more distal metastasis, as cancer cells elongate and take on more migratory phenotypes as they become more mesenchymal^82^. Conversely, the increase of OVCAR-3 spread area with CI-exosomes indicates more epithelial signatures and suggests that CI-exosomes may contribute to MET and local TME reorganization.

To further elucidate the differences in exosome populations, we characterized the physical and molecular properties of CI- and SEC- exosomes. Interestingly, physical properties, such as exosome diameter (Fig. 1A,B), and molecular profiles, such as miRNA make-up, were more heterogeneous in our SEC-exosomes (Fig. 2B) in comparison to CI-exosomes (Fig. 2B). The observed homogeny in CI-exosomes could be in part to the fact that CI-exosomes remain sequestered on the cell membrane until extracellular calcium is chelated to stimulate their release; whereas our SEC-exosome population includes all exosomes spontaneously released by cells over a 48-hour period. Previous work has shown that cells can use exosomes as autocrine signaling molecules^83^ and use cell adhesion molecules to dock and adhere to the plasma membranes^84^. It is well documented that cell adhesion molecules, including cadherins and integrins, are reliant on calcium signaling for activation^85^. Therefore, the downregulation of extracellular divalent cations could destabilize exosome attachments to the membrane and trigger their release. Thus, CI-exosomes could be a sub-population of SEC-exosomes released prematurely in response to perturbations in extracellular calcium.

Through biophysical functional assays, we were able to show that different exosome populations mediate functional cellular responses to direct the multistep metastatic process. These studies highlight that exosome heterogeneity, along with exosome miRNA content, contributes to the development of a more invasive EOC TME.

## Methods

### EOC cell culture

OVCAR-3 cells, obtained as a gift from Dr. John McDonald, were cultured in RPMI 1640 (Corning, USA) consisting of 10% Fetal Bovine Serum (FBS) (Atlanta Biologicals, USA), and 1% penicillin streptomycin (Corning, USA) and expanded in T-75 flasks (Thermo Fisher, USA). OVCAR-3 cells received changes of media every 3 days and were split at 80% confluence.

### CAF cell culture

CAFs were isolated from discarded EOC tissues collected from consented patients at Women & Infants Hospital and transferred to our lab as de-identified samples under Material Transfer Agreement with Women & Infants Hospital. The IRB protocol for this study was approved by the Women & Infants Hospital Institutional Review Board (IRB) committee and followed in accordance with federal, state, institutional, and ethical guidelines provided by the Office for Human Research Protections (OHRB) and U.S. Department of Health and Human Services (HHS). Written informed consent was obtained for this study from all participants. Tumor-derived fibroblasts were cultured in DMEM/F12 (Corning, USA), consisting of 10% FBS, 1% L-Glutamine, and 1% penicillin streptomycin and expanded in T-75 flasks. We performed flow cytometry for specific surface markers to confirm fibroblast phenotype according to previously published studies (Sup. Figure 1)^85,86]^. CAFs received changes of media every 3 days and were split at 80% confluence. Isolated CAFs were maintained for no more than 6 passages to minimize the effects of cellular senescence and population drift, which can occur with extended culture.

### Exosome collection

To harvest SEC-exosomes, serum-free cell culture media was collected after a 48-hour culture period with 70–90% confluent OVCAR-3 cells. Exosomes were collected using standard ultracentrifugation protocol^26^. The media was centrifuged at 2,000 × g for 20 minutes at 4 °C and at 10,000 × g for 30 minutes at 4 °C to remove cell debris; the remaining media was ultracentrifuged at 100,000 × g twice for 70 minutes to pellet exosomes. After ultracentrifugation, the exosome pellet was resuspended in 100 μl PBS and labeled with DiI (1,1′-dioctadecyl-3,3,3′,3′-tetramethylindocarbocyanin, Biotium, USA). To harvest CI-exosomes, cells were briefly washed with PBS to remove any residual media. OVCAR-3 cells were then treated with 10 mM of EDTA (Promega, USA) for 10 minutes in the incubator. The EDTA solution was subsequently collected and the CI-exosomes were harvested using the same standard ultracentrifugation methods to collect the SEC-exosomes. Briefly, the EDTA solution was centrifuged at 2,000 × g for 20 minutes and 10,000 × g for 30 minutes at 4 °C to remove any debris. The supernatant was then ultracentrifuged at 100, 000 × g twice for 70 minutes to pellet CI-exosomes. The CI-exosome pellet was subsequently resuspended in 100 μl PBS and labeled with DiI.

### Exosome treatment

CAFs were treated at an exosome concentration of 3.8 ×10^8^ exosomes/mL for each biophysical assay. CAFs were pretreated with either exosome population for 24 hours prior to experimentation.

### Dynamic light scattering (ZetaView)

Diluted exosome solution of 1:1000 was injected into the ZetaView Nanoparticle Tracking Video Microscope (Particle Metrix, Germany). A real-time video of the diluted exosome solution was monitored. Exosome diameter was determined using DLS methods.

### Immunoblotting

Equal number of CI- and SEC-exosomes were collected (counted using ZetaView) and immediately lysed. Lysed exosome samples were heated for 5 minutes and separated by sodium dodecyl sulfate polyacrylamide gel (SDS-PAGE) methods. Gels were then transferred onto polyvinylidene difluoride (PVDF) membranes (Thermo Fisher, USA) and probed using primary antibodies targeting for phospholipase A2 Group IIA (Abcam) and CD63 (Abcam). Bands were then visualized using Clarity ECL Western Substrate (BioRad, Hercules, CA, USA). Band intensities were measured and analyzed using Quantity One Analysis Software (BioRad).

### Exosome miRNA microarray

Total exosome RNA was isolated according to the manufacturer’s published protocol (Total Exosome RNA and Protein Isolation Kit, Invitrogen, USA). Briefly, exosomes were resuspended in the provided Exosome Resuspension Buffer. After acid-phenol chloroform extraction, the exosome RNA solution was washed several times and eluted through provided filter cartridges. Total RNA was also collected from OVCAR-3 cell lysates to serve as a control. 10 μg of exosome miRNA was labeled for Affymetrix array analysis using Encore Biotin Module (NuGEN Technologies, USA). The Genomics Core Facility at the Center for Genomics and Proteomics (Brown University, USA) carried out the array hybridization and analysis according to Affymetrix protocols.

### Exosome uptake studies

CAFs were seeded on 12 mm glass coverslips at 50% confluency. Fibroblasts were treated with or without CI- and SEC-exosomes for 24 hours. Cells were then fixed with 4% paraformaldehyde (PFA) and stained with DAPI. Coverslips were then mounted and fluorescence images were taken at 40X magnification using an inverted Nikon Eclipse Ti microscope.

### Dynasore treatment

CAFs were seeded on coverslips at 50% confluency and were treated with CI-exosome and SEC-exosome. Concurrently with exosome treatment, fibroblasts were treated with 10 nM of Dynasore (Selleckchem). CAFs were incubated with the exosome-dynasore solution for 24 hours and were fixed with 4% PFA and stained with DAPI. Coverslips were then mounted and fluorescence images were taken at 40X magnification using an inverted Nikon Eclipse Ti microscope.

### Flow cytometry

CAFs were treated with exosomes for one hour. Cells were detached, pelleted, suspended in FACS buffer (2% FBS and 1 mM EDTA in PBS), and fixed in 2% PFA. Samples were run on an easyCyte HT (Guava instruments) flow cytometer.

### Single-cell motility

CAFs were cultured to 20–40% confluency on wells coated with 20 ng/mL of fibronectin (FN) (Alfa Aesar, USA). Wells were coated with FN because there is increased deposition of FN in ovarian cancer ECM. Fibroblasts were pre-treated with exosomes for 24 hours before collecting time-lapsed cell migration videos. To track random cell motility, nuclei were labeled with Hoechst—a fluorescent dye used to stain DNA. An inverted Nikon Eclipse Ti microscope at 10X magnification was used to collect time-lapsed images of cells over a 12-hour period at 10-minute intervals. Individual cell tracks were analyzed from microscopy videos using custom-written MATLAB code.

### Quantitative real time-polymerase chain reaction (qRT-PCR)

Total RNA was isolated using Purezol (BioRad) following manufacturer’s recommendation. 1 μg of total RNA was converted to cDNA using the iScript cDNA synthesis kit (BioRad). cDNA was used for qRT-PCR to analyze the expression of genes listed in Sup. Table 3. Primers were obtained from IDT (USA). Relative expression values were normalized to 18 s and Control groups.

### Actin and Vinculin Immunocytochemistry

Cells were cultured at 50–60% confluency on FN coated coverslips. CAFs were then treated with CI- or SEC-exosomes for 24 hours. Cells were then fixed with 4% PFA, permeabilized with 0.5% Triton X-100 (Fisher Bioreagents, USA), blocked with 5% bovine serum albumin (BSA) (Alfa Aesar, USA), and stained with 1:200 anti-vinculin (Invitrogen, USA) in 2% BSA. CAFs were then washed and stained with 1:200 Rhodamine Phalloidin (Invitrogen, USA) and 1:500 of Alexa Flour 488 goat anti-rabbit (Invitrogen, USA). Cells were then visualized using an inverted Nikon Eclipse Ti to obtain fluorescence images at 40X and 100X magnification.

### Cell morphology

CAF shape factor was measured from 10X bright-field images. Data was quantified using ImageJ shape factor parameter defined as 4π*Area/Perimeter^2^. CAF shape factor of 1 and 0 represent a circle and line, respectively.

### Adhesion strength studies

CAFs were seeded on FN coated surfaces overnight and pre-treated with CI- or SEC-exosomes for 24 hours. Cells were then labeled with 1 µM Calcein-AM (BioLegends USA), a cell-permeant fluorescent dye. The CAF attachment fraction was measured using an established centrifugal force-based adhesion assay. Briefly, the supernatant (containing cell culture media and exosomes) was replaced with adhesion buffer before an initial fluorescence reading was taken. Plates were then inverted and centrifuged using a TS-5.1–500 rotor (Beckman Coulter) at 500 rcf for 5 minutes to remove loosely adherent cells before the final fluorescence reading. The attached fraction was reported as the final reading over the initial reading.

### Cell spreading studies

CAFs were cultured on FN coated surfaces to 50% confluency and were then pretreated with exosomes for 24 hours. Cells were then detached from surfaces, stained with Calcein-AM, and reseeded on FN coated surfaces. Images were taken every 30 minutes over a 12-hour period to analyze fibroblast-spreading profiles. Various cell shape parameters were measured using Image J. The cell shape factor was measured for the 12-hour time point.

### Coculture studies

OVCAR-3 cells were stained with 1 µM CFSE (BioRad, USA) according to manufacturer’s protocol. Equal number of CAFs and CFSE-labeled OVCAR-3 were mixed and seeded on surfaces. Coculture models were treated with or without exosomes. Time-lapsed 10X microscopy images were taken every 30 minutes over a 16-hour period to analyze various OVCAR-3 biophysical phenotypes. Initial and final cell shape factor and area measurements were done using ImageJ.

### Statistics

Data are reported as mean ± standard error of the mean (SEM). At least three experiments were performed for all biophysical experiments. Student’s t-test was calculated to determine experimental significance, where p < 0.05 was considered statistically significant (*p < 0.05, **p < 0.005, ##p < 0.005 ***p < 0.001). Experimental groups were compared to untreated (control) groups or time corresponding control groups. For normalized data, Kruskal-Wallis test was used to determine significance, where p < 0.05 was considered statistically significant. (*p < 0.05, **p < 0.005, ***p < 0.001).

## Supplementary information

Supplemental information.
