# Supplementary material for: Ovarian Cancer Exosomes Trigger Differential Biophysical Response in Tumor-Derived Fibroblasts
Source: Sci Rep. 2020 May 26;10:8686. doi: 10.1038/s41598-020-65628-3 (PMC7250846; doi:10.1038/s41598-020-65628-3)
Supplement: Supplementary file 1 — Supplemental information. [file 41598_2020_65628_MOESM1_ESM.pdf]

Ovarian Cancer Exosomes Trigger Differential Biophysical Response in Tumor-Derived Fibroblasts

Amy H. Lee[1], Deepraj Ghosh[2], Nhat Quach[2], Devin Schroeder[2], Michelle R. Dawson[*1,2]
1. Brown University, School of Engineering, Center for Biomedical Engineering, Providence, RI, 02912, USA.

2. Brown University, Department of Molecular Pharmacology, Physiology, and Biotechnology, Providence, RI, 02912, USA.

* Correspondence addressed to michelle_dawson@brown.edu

Supplemental Figure 1

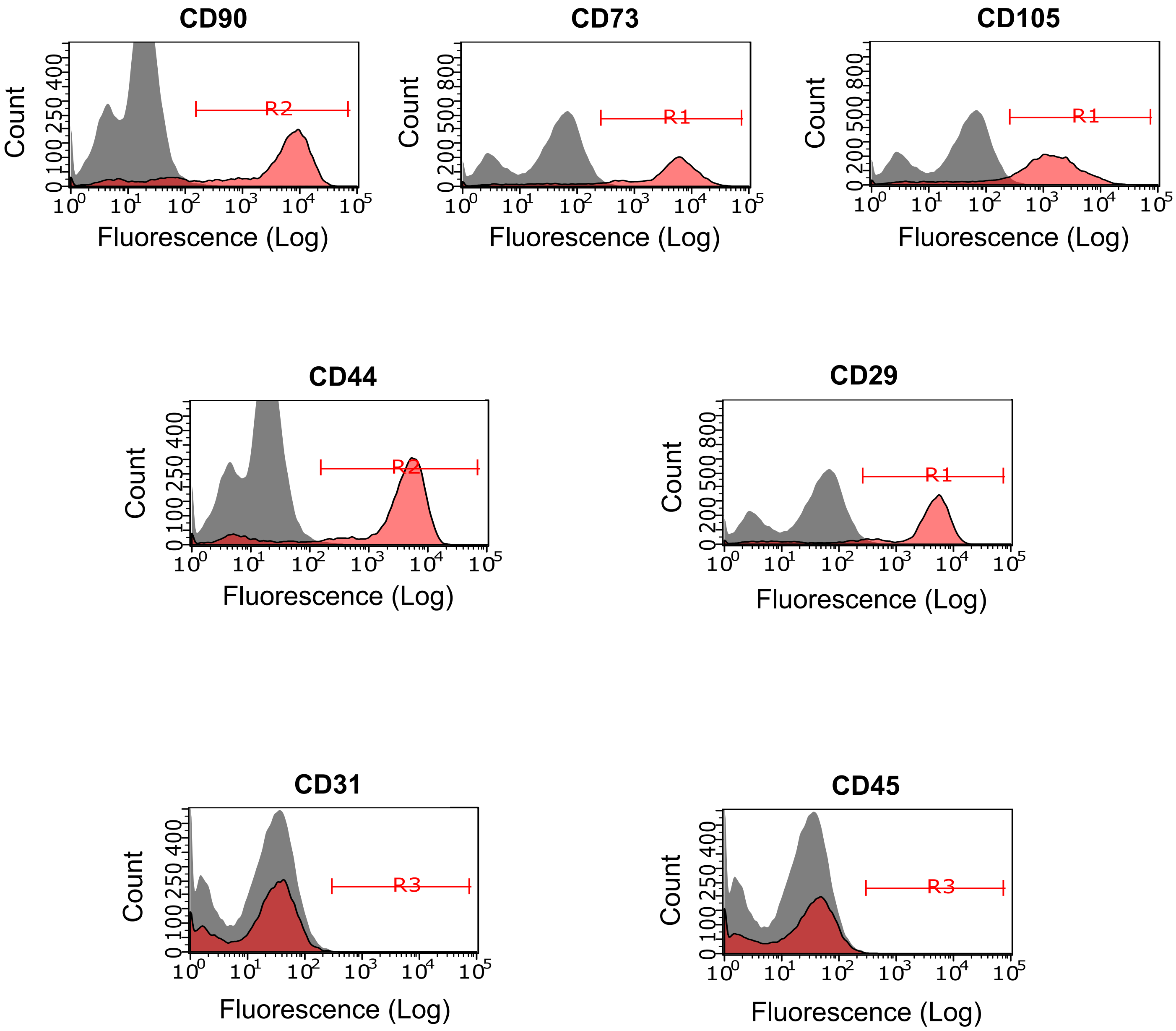

Supplemental Table 1

Pathways of Interest	# miRNAs	Involved Genes	Involved miRNAs
Proteoglycans in Cancer	261	CAMK2D, ACTB, ROCK1, SMAD2, CAV1, RHOA, AKT2, WNT5A, CD44	miR-7-1-3p, miR-940, miR-106b, let-7a-5p, let-7b-5p, let-7c-5p, let-7e-5p, let-7f-5p, let-7g-5p, let-7i-5p, miR-182-5p, miR-429, miR-195, miR-200b, miR-3613, miR-3714, miR-4470, miR-4728, miR-4668, miR-6768, miR-8063, miR-497, miR-513a-5p, miR-939-5p
Pathways in Cancer	233	PDGFRA, WNT16, TGFBR1, SMAD2, ROCK1, RHOA, CCNA1, Rac2, COL4A5, AKT2, MAPK3, FN1, CDK4, FOXO1, PTEN, JAK1, BRAF	miR-7-1-3p, let-7e-5p, let-7g-5p, let-7b-5p, let-7f-5p, let-7f-5p, miR-15a, miR-106b, miR-940, miR-182-5p, miR-200b, miR-429, miR-200c, miR-3613-3p, miR-3714, miR-1290, miR-6768-5p, miR-6876-5p, miR-939-5p, miR-664b-3p, miR-497, miR-513a-5p
TGF-B Signaling	154	ROCK1, SMAD2, SMAD6, RhoA, PPP2CA, SMURF2, ACVR2A, BAMBI	miR-7-1-3p, miR-106b-5p, miR-940, miR-224-3p, miR-429, miR-195-5p, miR-301a-3p, miR-3613-3p, miR-3149, miR-8063, miR-664b, miR-494-3p, miR-940, miR-522-3p, let-7a-5p, let-7b-5p, let-7c-5p, let-7d-5p, let-7e-5p, let-7f-5p, let-7g-5p, let-7i-5p
Wnt Signaling Pathway	249	FZD7, CTNNBIP1, CAMK2D, Wnt2B, Wnt16, RhoA, SMAD4, VANGL1, Rac2, CCND2	miR-7-1-3p, miR-15a-5p, miR-30b-5p, miR-30e-5p, miR-940, let-7a-5p, let-7b-5p, miR-26a-5p, has-miR195-5p, miR-182-5p, miR-205-5p, miR-224-3p, miR-3613-3p, miR-3619-5p, miR-4668-5p, miR-6124, miR-6876-5p, miR-6768-5p, miR-664b-3p, miR-497-5p, miR-513a-5p
Ras Signaling Pathway	274	RASAL1, PRKCA, PDGFRA, KRAS, PAK1, FGF11, RAB5A, Rac2, PIK3CB, Akt3, FZD6, RhoA, MAPK9, CAMK2D	miR-7-1-3p, miR-30e-5p, miR-106b-5p, miR-15a-5p, miR-155-5p, miR-200b-3p, miR-224-3p, miR-429, miR-182-5p, miR-195-5p,miR-3613-3p, miR-1290, miR-3149, miR-6124, miR-4668-5p, miR-4728-5p, miR-4470, miR-6768-5p, miR-6756-5p, miR-8063, miR-8089, miR-7111, miR-513a-5p, miR-664b-3p, miR-497-5p, let-7a-5p, let-7b-5p, let-7c-5p, let-7d-5p, let-7e-5p, let-7f-5p, let-7g-5p, let-7i-5p
Focal Adhesions	261	ACTB, MYLK4, Rock1, COL4A5, RAF1, RhoA, FYN, CAV3, VCL, PAK3, ACTN1, PARVG, ITGA2, FN1, PXN	miR-15a-5p, miR-8075, miR-7-1-3p, miR-940, let-7a-5p, miR-30e-5p, miR-429, miR-195-5p, miR-182-5p, miR-200b-3p, miR-3613-3p, miR-1290, miR-4728-5p, miR-4470, miR4668-5p, miR-6124, miR-6768-5p, miR6756-5p, miR-6165, miR-6808-5p, miR-8063, miR-664b-3p, miR-513a-5p, miR-497-5p, miR-1207-5p
Adherens Junction	233	ACTN2, SMAD2, SNAI2, RHOA, CDH1, CTNND1, PVRL4, MAPK1, RAC1, WASF1	miR-7-1-3p, miR-940, miR-182-5p, miR-205-5p, miR-3613-3p, miR-3714, miR-4668-5p, miR-4728-5p, miR-6893-5p, miR-6808, miR-8063, miR-940, miR-589-3p, let-7a-5p, let-7b-5p, let-7e-5p
Regulation of Actin Cytoskeleton	199	ARPC5, PFN1, ACTN2, PGGFRA, MYLK4, VCL, MYH9, KRAS, FGF2, PAK6, FN1, ROCK2, PXN	miR-182-5p, miR-224-3p, miR-429, miR-200b-3p, miR-195-5p, miR-3613-3p, miR-3714, miR-1290, miR-4668-5p, miR-4728-5p, miR-4689, miR-6124, miR-5196-5p, miR-6768-5p, miR-6876-5p, miR-6756-5p, miR8063, miR-8089, miR-8075, miR-664b-3p, miR-513a-5p, miR-940, miR-497-5p

Supplemental Figure 2

CI-Exosomes vs. Cell Lysates

A.

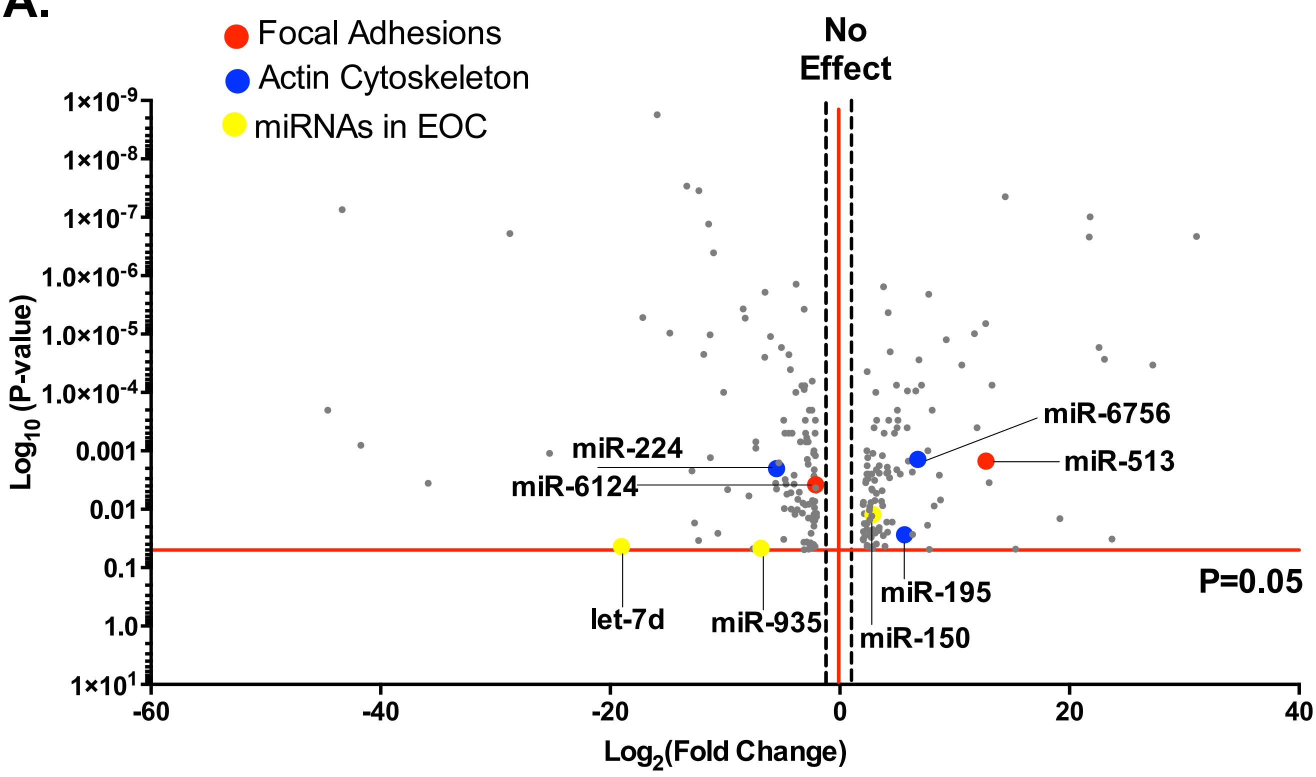

SEC-Exosomes vs. Cell Lysates

B.

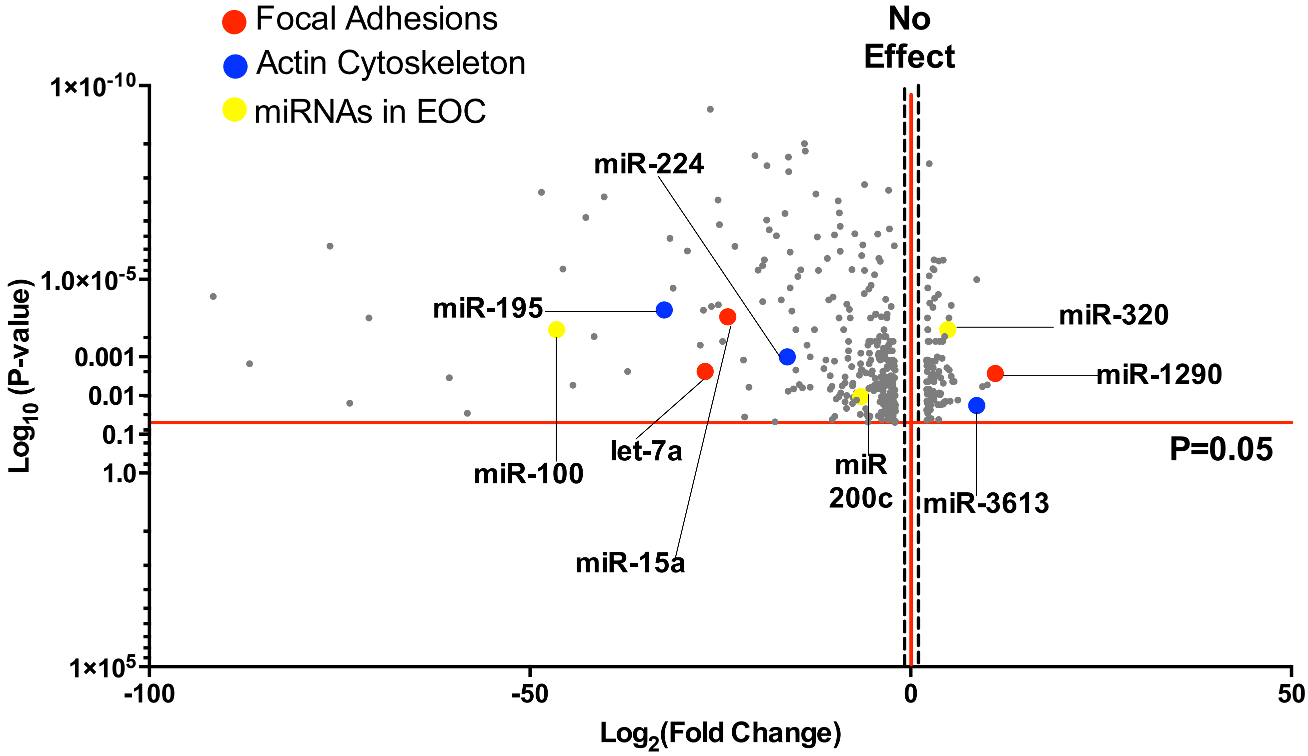

Supplemental Figure 3

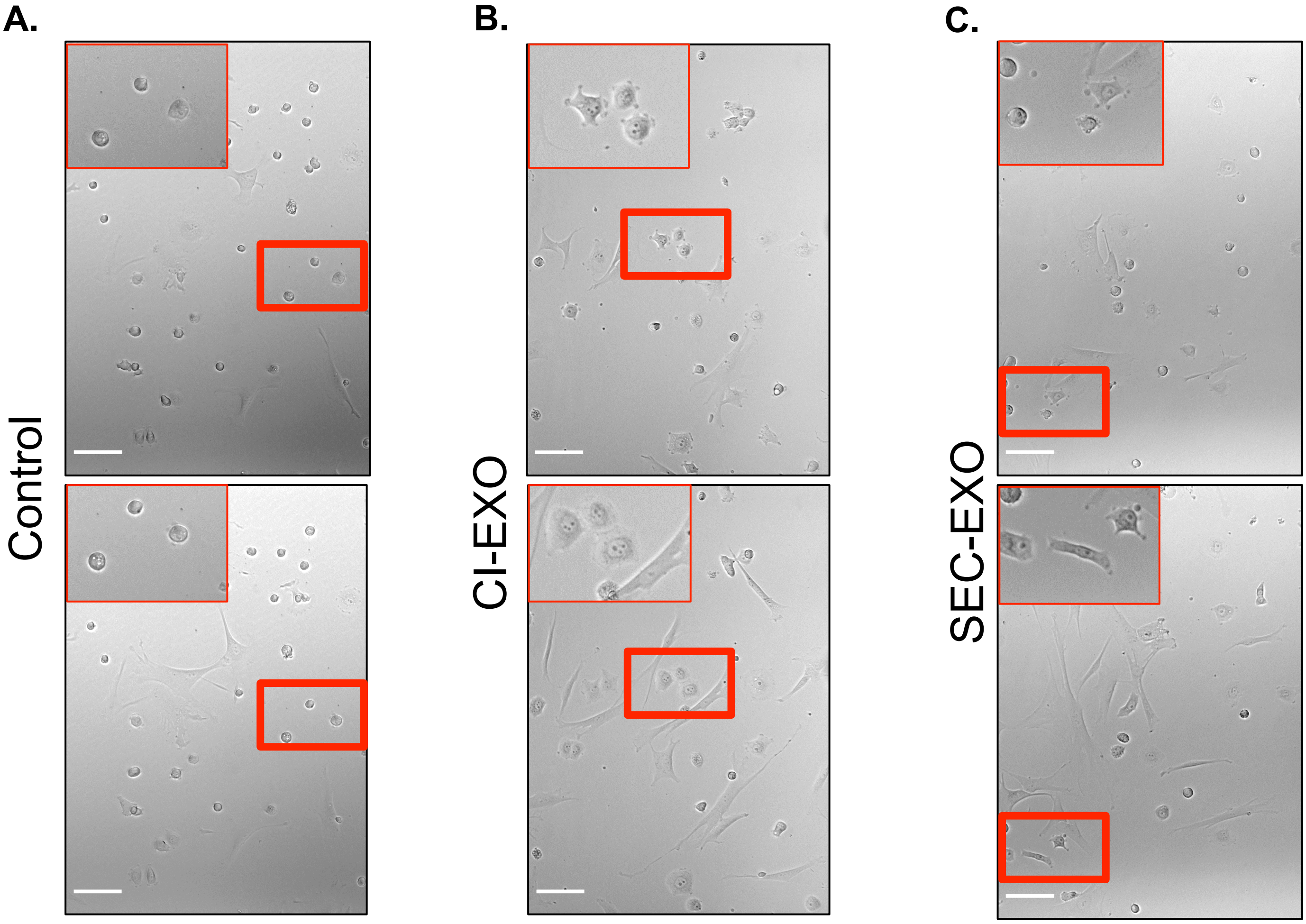

Supplemental Table 2:

A.

	Mean	Median	CV
Control (I)	0.807192	0.833	0.126
Control (F)	0.760992	0.761	0.144
CI-EXO (I)	0.768791	0.7945	0.145
CI-EXO (F)	0.81214	0.826	0.104
SEC-EXO (I)	0.790073	0.8195	0.132
SEC-EXO (F)	0.714079	0.763	0.238

Supplemental Figure 4

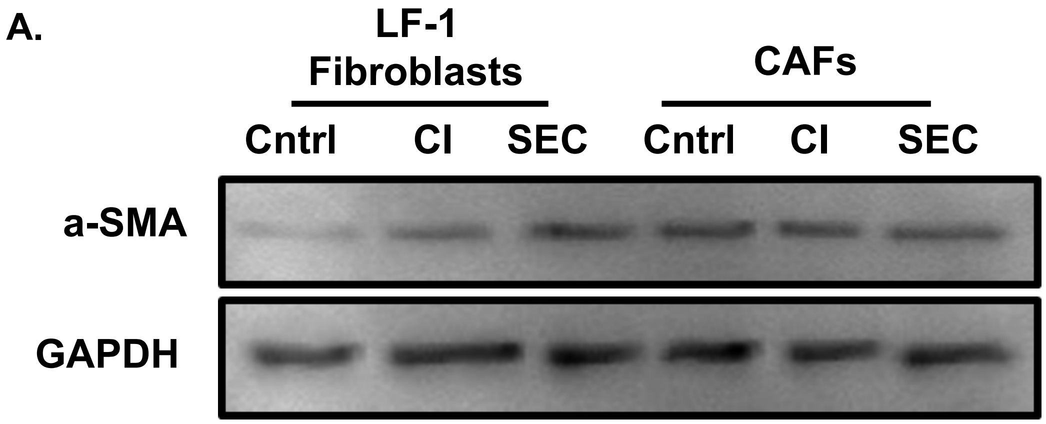

Supplemental Figure 5

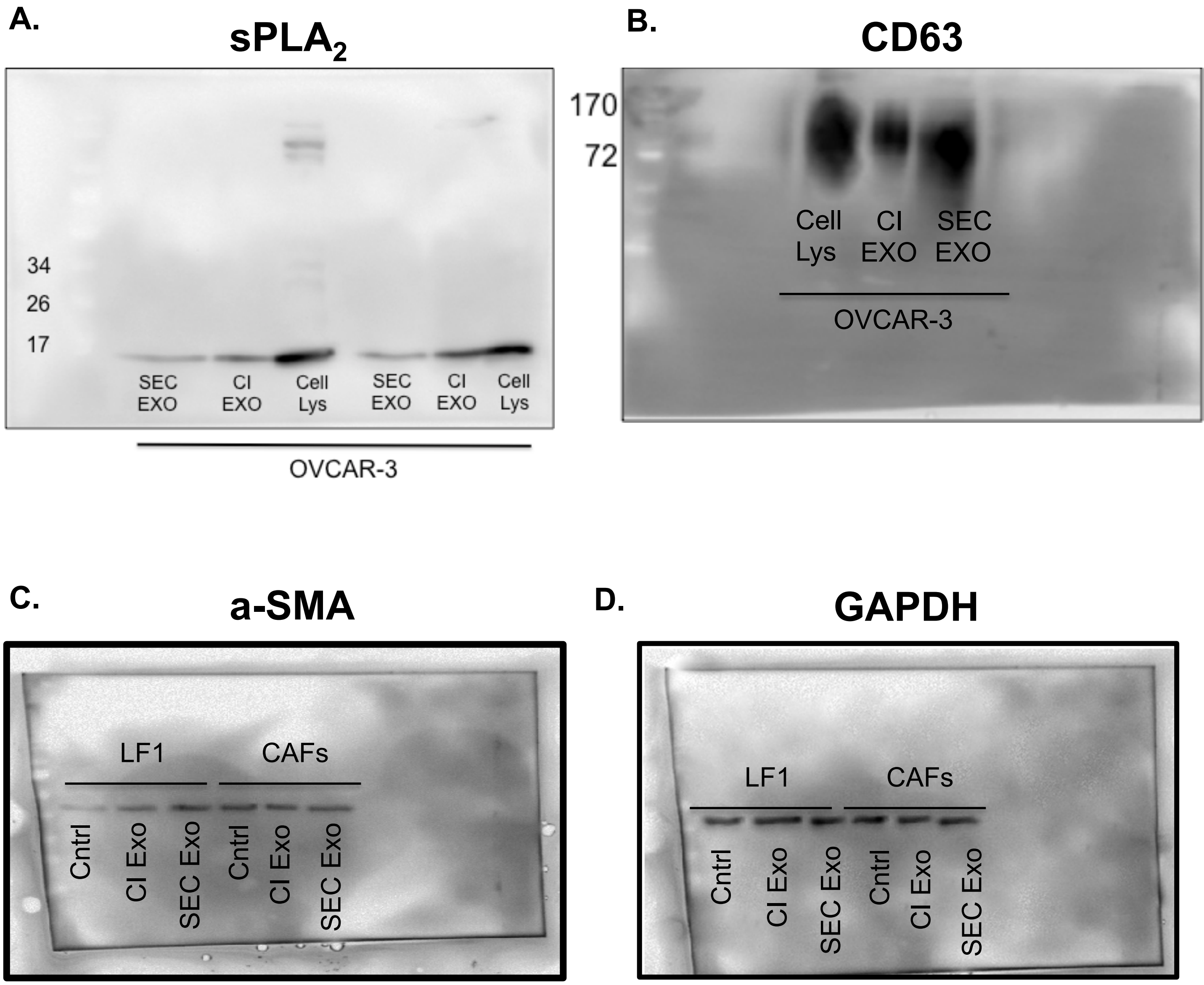

Supplemental Table 3:

MYL9	F: ACC CCA CAG ACG AAT ACC TG
	R: AAA GGC GTT GCG AAT CAC AT
RHOA	F: TAT CGA GGT GGA TGG AAA GC
	R: TAT CGA GGT GGA TGG AAA GC
ROCK1	F: TTA CTG ACA GGG AAG TGA GGT T
	R: AGG TAG TTG ATT GCC AAC GAA A
ROCK2	F: AAC AGG CAT GGT ACA TTG TGA T
	R: GGA AAA CAC CTA CAG ACC ACC
18s	F: GTA ACC CGT TGA ACC CCA TT
	R: CCA TCC AAT CGG TAG TAG CG
